# Enhancing diagnostic precision in EBV-related HLH: a multifaceted approach using ^18^F-FDG PET/CT and nomogram integration

**DOI:** 10.1186/s40644-024-00757-w

**Published:** 2024-08-18

**Authors:** Xu Yang, Xia Lu, Lijuan Feng, Wei Wang, Ying Kan, Shuxin Zhang, Xiang Li, Jigang Yang

**Affiliations:** 1 Department of Nuclear Medicine, Beijing Friendship Hospital, Capital Medical University, 95 Yong An Road, Xi Cheng District, Beijing, 100050 China; 2 Division of Nuclear Medicine, Department of Biomedical Imaging and Image-guided Therapy, Vienna General Hospital, Medical University of Vienna, Währinger Gürtel 18-20, Wien, Vienna, 1090 Austria

**Keywords:** Epstein-Barr virus, Hemophagocytic lymphohistiocytosis, Lymphoma, ^18^F-FDG PET/CT, Nomogram

## Abstract

**Background:**

The hyperinflammatory condition and lymphoproliferation due to Epstein-Barr virus (EBV)-associated hemophagocytic lymphohistiocytosis (HLH) affect the detection of lymphomas by ^18^F-FDG PET/CT. We aimed to improve the diagnostic capabilities of ^18^F-FDG PET/CT by combining laboratory parameters.

**Methods:**

This retrospective study involved 46 patients diagnosed with EBV-positive HLH, who underwent ^18^F-FDG PET/CT before beginning chemotherapy within a 4-year timeframe. These patients were categorized into two groups: EBV-associated HLH (EBV-HLH) (*n* = 31) and EBV-positive lymphoma-associated HLH (EBV + LA-HLH) (*n* = 15). We employed multivariable logistic regression and regression tree analysis to develop diagnostic models and assessed their efficacy in diagnosis and prognosis.

**Results:**

A nomogram combining the SUVmax ratio, copies of plasma EBV-DNA, and IFN-γ reached 100% sensitivity and 81.8% specificity, with an AUC of 0.926 (95%CI, 0.779–0.988). Importantly, this nomogram also demonstrated predictive power for mortality in EBV-HLH patients, with a hazard ratio of 4.2 (95%CI, 1.1–16.5). The high-risk EBV-HLH patients identified by the nomogram had a similarly unfavorable prognosis as patients with lymphoma.

**Conclusions:**

The study found that while ^18^F-FDG PET/CT alone has limitations in differentiating between lymphoma and EBV-HLH in patients with active EBV infection, the integration of a nomogram significantly improves the diagnostic accuracy and also exhibits a strong association with prognostic outcomes.

**Supplementary Information:**

The online version contains supplementary material available at 10.1186/s40644-024-00757-w.

## Key points

• In EBV-HLH, a multivariate nomogram with EBV-DNA, IFN-γ, and SUVmax-LN/M significantly enhances FDG PET/CT efficacy in lymphoma diagnosis.

• The multivariate nomogram also predicted prognosis, and high-risk EBV-HLH had a similar prognosis to EBV-positive lymphoma-associated HLH.

**Supplementary Information:**

The online version contains supplementary material available at 10.1186/s40644-024-00757-w.

## Introduction

HLH is a lethal systemic inflammatory disorder, arising from the interplay of genetic and exposure factors. It is characterized by the hyperactivation of cytotoxic T cells, natural killer cells and macrophages, resulting in a profound cytokine storm [1]. Adults account for approximately 40% of HLH patients, with an estimated incidence of about 1 in 800,000 [2]. At tertiary medical centers, the prevalence is projected to be approximately 1 in every 2000 adult admissions [3, 4]. HLH is typically classified into primary or familial HLH, and secondary HLH (sHLH), contingent upon the detection of HLH-predisposing genetic abnormalities. Primary HLH is predominantly identified in pediatric patients, while the majority of cases in adults are sHLH. sHLH is primarily associated with malignancy, infections, and rheumatologic disorders. The malignancy predominantly encompasses haematological malignancies, particularly lymphoma. Lymphoma and EBV represent the most prevalent causes, albeit their proportions vary geographically, ranging from 32 to 45% for lymphoma and 15–33% for EBV [2, 3, 5]. EBV has overtaken lymphoma as the most common cause of sHLH in some East Asian studies. The proportion of lymphomas associated HLH increase progressively with age [3]. Notably, EBV viremia sometimes coexist with lymphoma [6].

The presence of lymphomas in sHLH patients is a critical factor that determines the need for lymphoma-specific therapy and directly impacts prognosis. Detecting underlying lymphomas is therefore of utmost importance. Clinical features and laboratory abnormalities of lymphoma often overlap with those of HLH, making identification challenging. Some markers like soluble IL2 receptor/ferritin, interferon (IFN)-inducible protein 10/CXCL10, and monokine-induced by IFN-γ/CXCL9 have been proposed to aid in diagnosing lymphoma-associated HLH (LA-HLH), but their accuracy lacks prospective validation [7–9]. Lymphoma diagnosis requires tissue biopsy, which presents challenges in terms of site identification, invasiveness, and prolonged result waiting times, all in contrast to the rapid deterioration of HLH to multi-organ failure and death.

^18^F-FDG PET/CT, as whole-body metabolic imaging, has been recommended as a valuable tool for suspected HLH patients, aiding in the detection of malignancies, such as lymphoma, and guiding further biopsies [10]. Notably, LA-HLH patients exhibit higher FDG uptake in the liver, spleen, bone marrow, and lymph nodes compared to non-malignancy-associated HLH patients, and good diagnostic accuracy can be achieved though integrating clinical parameters [11]. However, several reports have noted that the ^18^F-FDG PET/CT findings of patients with EBV-HLH closely resemble those of lymphoma patients, with focal or diffuse increased FDG uptake in the spleen and bone marrow, along with enlarged lymph nodes displaying elevated FDG uptake, especially in EBV-associated lymphoproliferative disorders (LPD). In individuals with active EBV infection, ^18^F-FDG PET/CT is ineffective for identifying lymphoma [12–14].

The aim of this study was to explore the efficacy of ^18^F-FDG PET/CT in distinguishing lymphomas among HLH patients with active EBV infection. This was achieved by integrating ^18^F-FDG PET/CT parameters with clinical variables, and disease outcomes serving as an external validation measure.

## Methods

### Patients

The study received Institutional Review Board (IRB) approval (BFHHZS20230088), and informed consent was obtained over the telephone from all individual participants included in the study. This retrospective study included all consecutive patients with HLH who underwent ^18^F-FDG PET/CT in department of nuclear medicine in Beijing Friendship Hospital between January 2018 and July 2022. The inclusion criteria were as follows: (1) HLH diagnosis was made based on the HLH-2004 criteria [15]; (2) age over 18 years; (3) confirmation of EBV infection through detection of EBV-encoded small RNA (EBER) using immunohistochemistry staining of biopsy tissue and/or quantification of EBV-DNA copy number via real-time PCR from the patients’ blood; (4) completion of ^18^F-FDG PET/CT prior to induction chemotherapy. Additionally, only sHLH patients with EBV-associated HLH (EBV-HLH) and lymphoma-associated HLH (LA-HLH) were included, while secondary causes such as plasma cell disease, solid tumors, and rheumatologic disorders like Still’s disease were excluded in this study. The diagnosis of lymphoma and determination of its pathological type were based on the WHO 2016 criteria for hematopoietic malignancies [16]. Primary HLH patients were excluded through Sanger or next-generation sequencing. Patients who had received granulocyte colony-stimulating factor within 1 week before the ^18^F-FDG PET/CT and those who did not undergo a biopsy for pathological diagnosis during the follow-up period were excluded. Additionally, patients with poor PET image quality due to factors such as high physiological muscle uptake were also excluded. Patients diagnosed with lymphoma by bone marrow aspirate or tissue biopsy were categorized into the EBV + LA-HLH group, whereas patients without malignancy detection were assigned to the EBV-HLH group.

### Patient record review and follow-up

Relevant clinical characteristics and laboratory results were reviewed from the electronic medical records of Beijing Friendship Hospital. The highest temperature of patients in the 24 h prior to the PET scan was recorded. The laboratory data were restricted to the 2-week period before and after the PET scan, with a preference for the most recent tests preceding the scan. The collected laboratory parameters included blood routine tests, inflammatory markers, blood biochemical indexes, factors indicating immune response, cytokines, and EBV-related examinations. The presence or absence of hemophagocytosis and gene rearrangements in bone marrow were recorded. All patients were followed by telephone for at least 1 year after the PET scan, and overall survival (OS), defined as the time between the PET scan and death from any cause, was recorded.

### ^18^F-FDG PET/CT imaging and analysis

All combined PET/CT scans were conducted on a Siemens Biograph mCT scanner (Siemens Healthineers), according to the European Association of Nuclear Medicine (EANM) guidelines version 2.0 [17]. Patients were required to fast for a minimum of 6 h before the scan, and their glucose levels needed to be below 11.1 mmol/L at the time of tracer injection. PET/CT data acquisition occurred 60 ± 10 min after intravenous injection of 4.44 MBq/kg of ^18^F-FDG. All ^18^F-FDG-PET/CT images were retrospectively reviewed by two experienced nuclear medicine physicians and supervised by another nuclear medicine specialist. They were blinded to any clinical information. The long and short diameters of abnormal lymph nodes, the long diameter of the spleen, and the serous effusion were documented. Elliptical volume of interests (VOIs) were meticulously delineated, separately covering the entire lymph nodes, bone lesions, and other extranodal lesions. The hypermetabolic lymph nodes in the upper jugular region (cervical II), mediastinum, and hilum were excluded from measurement due to inflammatory hyperplasia, unless a lymphomatous lesion was considered. Additionally, it was essential to exclude FDG uptake in bone lesions resulting from degeneration, fractures, and bone penetration. For bone marrow SUVmax measurement, prioritize vertebrae without focal hypermetabolic lesions. If an L4 vertebra had such a lesion, the L3 vertebra was chosen, followed by the L5 and L2 vertebrae. As a reference value, the SUVmax of mediastinum was measured by placing a spherical VOI with a 1 cm diameter in the center of the descending aorta lumen. The ratio was calculated by dividing the SUVmax of the lesion or organs by that of the mediastinum.

### Statistical analysis

Statistical analyses were conducted using SPSS statistical software (version 27.0, IBM Corp.), MedCalc statistical software (version 20.027, MedCalc Software bvba), and R (version 4.2.3, http://www.r-project.org). Descriptive analyses included medians (interquartile ranges) or means ± standard deviations for skewed or normally continuous variables, and frequencies with percentages for categorical variables. To compare variables between the two group, the Mann-Whitney U test or t-test was applied for skewed or normally continuous variables, and Pearson’s chi-square (χ^2^) test or Fisher’s exact test was employed for categorical variables. Paired Spearman correlation coefficients between the ^18^F-FDG PET/CT parameters and clinical variables were calculated. A two-sided significance level of *p* < 0.05 was considered statistically significant for all tests.

We generated a decision tree using classification and regression analysis (CART), screened for variables that exhibited statistical differences between the two groups. To avoid overfitting due to the small sample, 10-fold cross validation was performed. Subsequently, we conducted multivariable binary logistic regression analyses employing the selected variables to develop laboratory, FDG PET, and combined models, respectively. The nomograms were constructed to visualize these models. Calibration curves and decision curve analysis (DCA) were employed to assess their predictive agreement and clinical utility.

Receiver-Operating-Characteristic (ROC) curves were utilized to access the diagnostic efficacy of the variables and models. We determined the optimal cutoff point for each variable or for the predicted probability of the models based on the highest Youden index. Delong’s test was applied to compare the area under the curve (AUC) of the models. The Net Reclassification Index (NRI) and Integrated Discrimination Improvement (IDI) between the models were calculated to evaluate the models’ reclassification improvement.

**Fig. 1:**
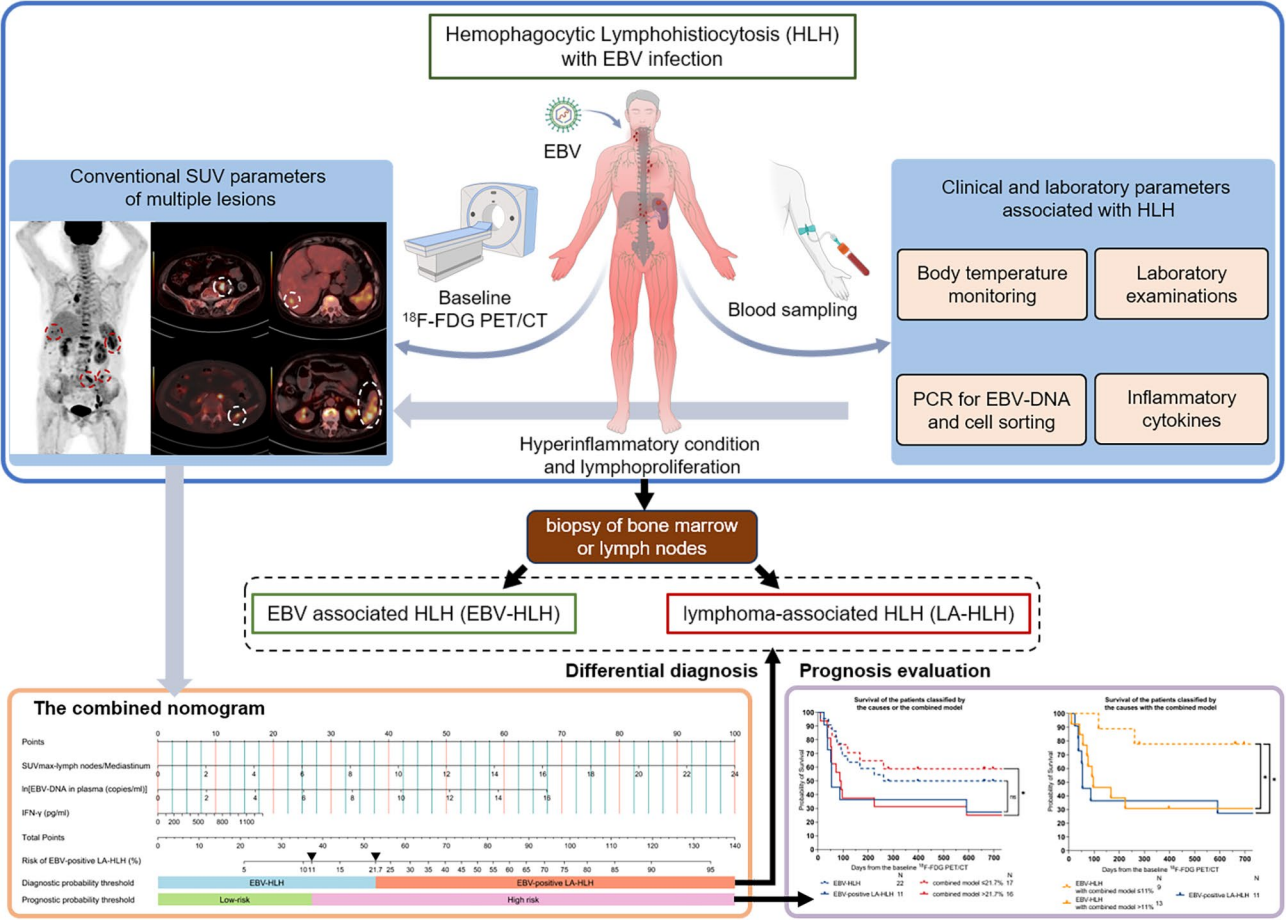
The visual abstract or workflow of this research

Finally, patients with different etiologies were further grouped based on the combined model. Survival curves were plotted using the Kaplan-Meier method, and group differences were analyzed using the log-rank test to evaluate the prognostic value of the model. Running log-rank tests were used to determine the cut-off probability of the model for predicting prognosis. The hazard ratios (HRs) and their 95% confidence intervals (CIs) for mortality were determined using the Mantel-Haenszel test. Our workflow is shown in Fig. 1.

## Results

### Patient characteristics

**Fig. 2:**
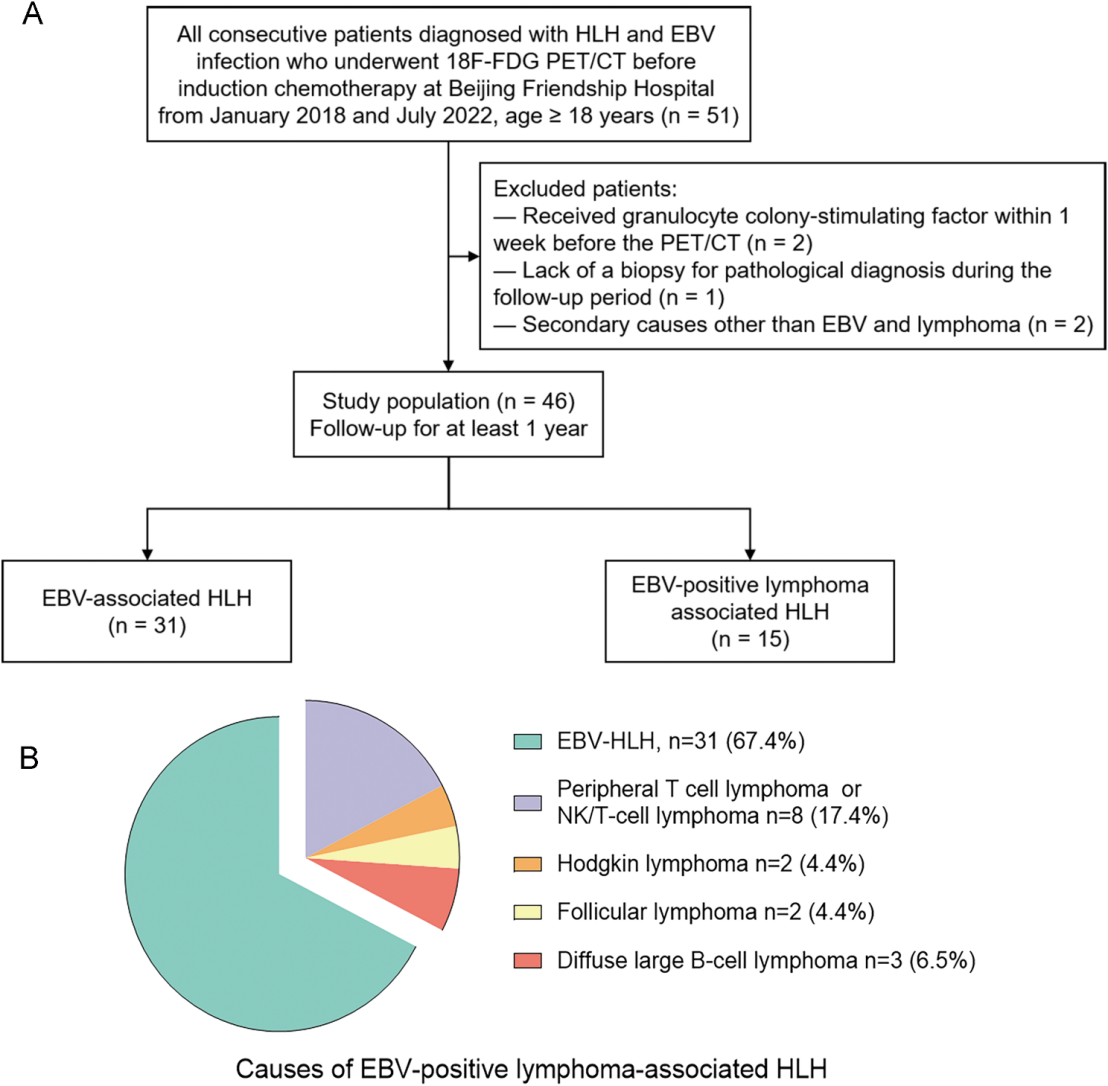
Patient inclusion flow chart (**A**) and pie chart showing the proportion of enrolled patients (**B**)

46 patients were included in the study, as depicted in Fig. 2A. Subsequently, following a 1-year follow-up, 15 patients were diagnosed with EBV-positive LA-HLH, while 31 patients were diagnosed with EBV-HLH based on pathological findings. The distribution of lymphoma subtypes was shown in Fig. 2B. The general characteristics were compared in Table 1. In the cohort of adult patients with HLH and EBV infection, the incidence of lymphoma was found to be higher in males, although no statistical significance between the two genders. Pathological EBER positivity on biopsy of bone marrow or lymph nodes was more frequent in EBV-positive LA-HLH. Clonal TCRB rearrangement was detected in one patient with EBV-HLH, while clonal IGH rearrangement was detected in one patient with EBV-positive follicular lymphoma-associated HLH. Flow cytometric sorting demonstrated that 90.6% of patients had EBV-infected T, NK/NKT, or B cells detected in their peripheral blood, with no statistically significant difference in the positivity rates of each lymphocyte subpopulation between the EBV-HLH and EBV-positive LA-HLH groups.

**Table 1:** General characteristics of adult HLH patients with EBV infection

Variables	Total, *n* = 46	EBV-HLH, *n* = 31	EBV-positive LA-HLH, *n* = 15	*P* value
General				
Female/Male, N (%)	17 (37.0)/29 (63.0)	13 (41.9)/18 (58.1)	4 (26.7)/11 (73.3)	0.315
Age, year, median (25%, 75%)	42.5 (26.75, 57.5)	44 (27, 60)	42 (26, 52)	0.752
Pathological findings				
Hemophagocytosis, N (%)	27 (60), *n* = 45	18 (60), *n* = 30	9 (60), *n* = 15	1.000
EBER, N (%)	23 (57.5), *n* = 40	11 (44), *n* = 25	12 (80), *n* = 15	0.026^*^
Gene rearrangement (Positive)^†^, N (%)	2 (6.3), *n* = 32	1 (5), *n* = 20	1 (8.3), *n* = 12	1.000
EBV-infected lymphocytes subsets^‡^, N (%)	29 (90.6), *n* = 32	21 (91.3), *n* = 23	8 (88.9), *n* = 9	
CD3 + CD4 + T cells	9 (28.1)	6 (26.1)	3 (33.3)	0.685
CD3 + CD8 + T cells	10 (31.3)	9 (39.1)	1 (11.1)	0.210
CD56 + NK/NKT cells	19 (59.4)	13 (56.5)	6 (66.7)	0.704
CD3- CD19 + B cells	21 (65.6)	15 (65.2)	6 (66.7)	1.000
T or NK cells	22 (68.8)	16 (69.6)	6 (66.7)	1.000

^†^The positive of gene rearrangement included immunoglobulin H (IGH), immunoglobulin kappa (IGK), immunoglobulin lambda (IGL), T cell receptor beta (TCRB), T cell receptor delta (TCRD), or T cell receptor gamma (TCRG) gene rearrangement

^‡^Flow cytometric sorting was used and EBV DNA was quantified by real-time fluorescence quantitative PCR (qPCR) in peripheral blood samples

EBV = Epstein-Barr virus; EBER = EBV-encoded RNAs; NK = natural killer;

Data are median (25%, 75%), or number(%)

^*^Significance at *P* < 0.05

### ^18^F-FDG PET/CT analysis

**Fig. 3:**
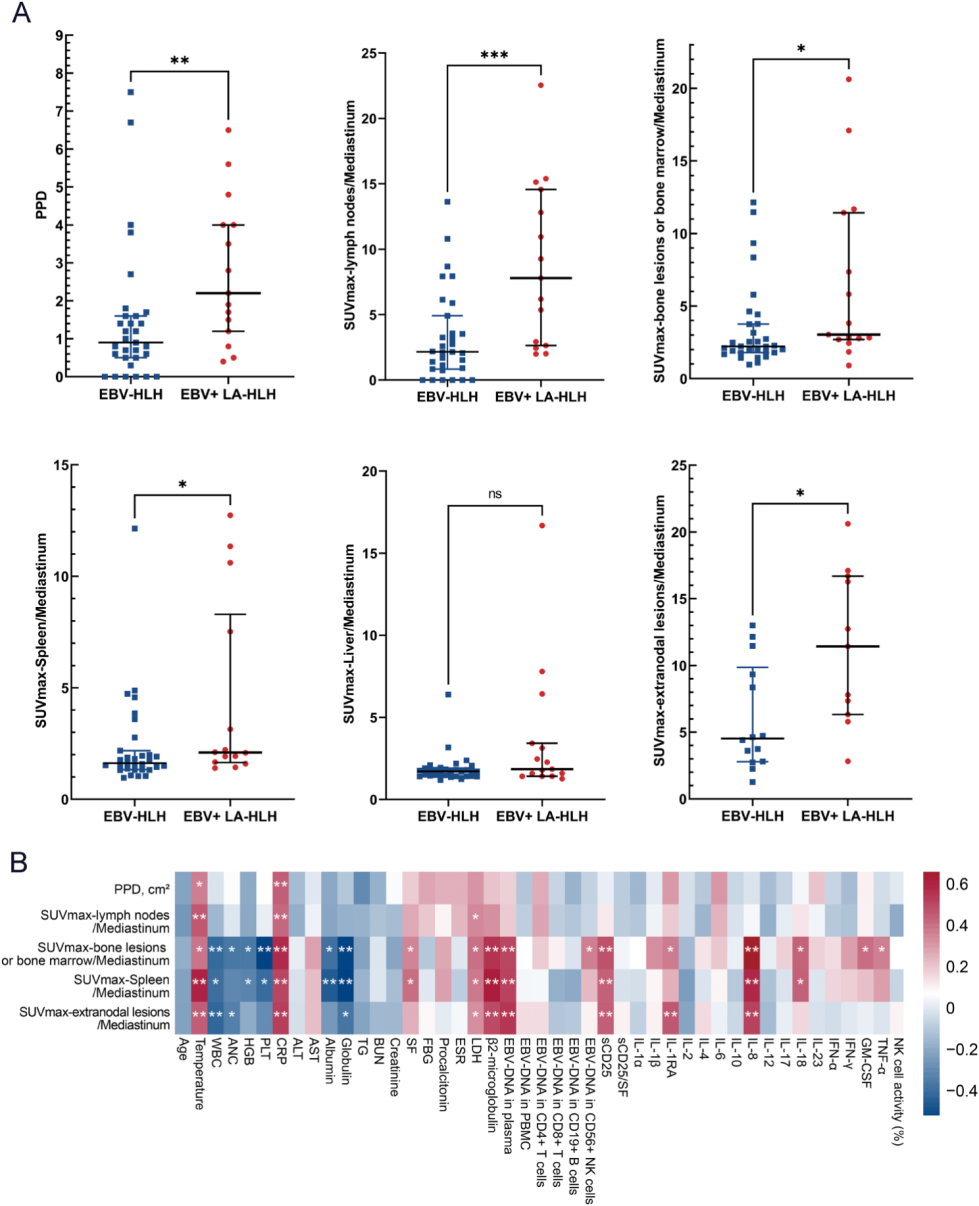
Comparison of key baseline ^18^F-FDG PET/CT parameters between the two groups and correlation analysis. Comparison of key baseline ^18^F-FDG PET/CT parameters between EBV associated HLH (EBV-HLH) and EBV-positive lymphoma-associated HLH (LA-HLH) (**A**) and the pairwise correlation between the ^18^F-FDG PET/CT parameters and clinical variables (**B**)

The ^18^F-FDG PET/CT parameters were compared in Table 2. Serous effusion is a common imaging manifestation of HLH and did not show significant differences between EBV-HLH and EBV-positive LA-HLH patients. Extranodal lesions were observed in the both groups, and there was no statistically significant difference in their frequency. The extranodal organs involved, in descending order of occurrence, were bone (21 cases), spleen (9 cases), liver (3 cases), subcutaneous tissue (3 cases), and other organs, each observed in 1 case, including nasopharynx, oropharynx, parotid gland, lung, stomach, and pancreas. Lymph nodes were significantly larger in patients with EBV-positive LA-HLH than in patients with EBV-HLH. However, the long diameter of spleen was not statistically different between the two groups. In patients with EBV-positive LA-HLH, lymph nodes, spleen, bone lesions or bone marrow, and other extranodal lesions exhibited significantly higher SUVmax ratios to the mediastinum compared to those with EBV-HLH. However, there were substantial overlaps between the two groups, as shown in Fig. 3A.

**Table 2:** Baseline 18 F-FDG PET/CT findings in adult HLH patients with EBV infection

Variables	EBV-HLH (*n* = 31)	EBV-positive LA-HLH (*n* = 15)	*P* value
Image finding, N (%)			
Serous effusion	15 (48.4)	8 (53.3)	0.753
Hydrothorax	8 (25.8)	8 (53.3)	0.066
Ascites	12 (38.7)	5 (33.3)	0.723
Polyserositis	5 (16.1)	5 (33.3)	0.345
Lymph node features			
High FDG-avid lymph nodes	25 (80.6)	15 (100)	0.174
LDi, cm	1.3 (0.8, 1.6)	1.9 (1.4, 2.5)	0.010^*^
SDi, cm	0.7 (0.5, 1.1)	1.4 (0.8, 1.6)	0.008^**^
PPD, cm^2^	0.86 (0.50, 1.60)	2.24 (1.21, 4.00)	0.008^**^
SUVmax-lymph nodes/Mediastinum	2.1 (0.8, 4.9)	7.8 (2.6, 14.6)	0.001^**^
Spleen and liver features^†^			
Spleen long diameter	14.1 ± 4.8 (*n* = 31)	15.0 ± 4.5 (*n* = 14)	0.562
SUVmax-Spleen/Mediastinum	1.6 (1.3, 2.2) (*n* = 31)	2.1 (1.6, 8.3) (*n* = 14)	0.027^*^
SUVmax-Liver/Mediastinum	1.7 (1.4, 1.9)	1.8 (1.4, 3.4)	0.114
Bone features			
focal bone lesion	12 (38.7)	9 (60.0)	0.174
SUVmax-bone lesion/Mediastinum	4.5 (3.0, 9.1) (*n* = 12)	7.4 (3.3, 14.4) (*n* = 9)	0.219
SUVmax-bone marrow/Mediastinum	2.0 (1.8, 2.5)	2.3 (2.2, 2.8)	0.256
SUVmax-bone lesions or bone marrow/Mediastinum	2.2 (1.8, 3.8)	3.0 (2.7, 11.4)	0.025^*^
Extanodal lesions			
Extranodal lesion, positive	14 (45.2)	11 (73.3)	0.072
Extranodal lesions except in bone	8 (25.8)	6 (40.0)	0.523
Extranodal lesions in multiple organs^‡^	6 (19.4)	5 (33.3)	0.501
SUVmax-extranodal lesions	6.7 (4.3, 14.9) (*n* = 14)	14.5 (9.7, 19.8) (*n* = 11)	0.009^**^
SUVmax-extranodal lesions/Mediastinum	4.5 (2.8, 9.9) (*n* = 14)	11.4 (6.3, 16.7) (*n* = 11)	0.018^*^

^†^One patient with EBV positive LA-HLH infection had been removed spleen, the number of spleen parameters minus 1 is 14. (*n* = 14). ^‡^When the extranodal lesions involved more than one organ. LDi = longest transverse diameter; SDi = shortest axis perpendicular to LDi; PPD = Products of the longest perpendicular diameters

Data are mean ± SD, median (25%, 75%), or number(%). *Significance at *P* < 0.05. **Significance at *P* < 0.01

### Baseline laboratory examinations

The temperature, serum β2-microglobulin levels, serum IFN-γ levels, and EBV DNA copies in plasma were significantly higher in EBV-positive LA-HLH patients compared to EBV-HLH (Supplement Table 1). Additionally, as depicted in Fig. 3B, there is a positive correlation between the size and metabolism of lymph nodes with body temperature and C-reactive protein levels. Furthermore, the metabolism of bone lesions or bone marrow, as well as the spleen, exhibited a stronger correlation with laboratory examinations. Specifically, body temperature, levels of C-reactive protein, serum β2-microglobulin, serum sCD25, and IL-8, as well as EBV DNA copies in plasma, showed a significant positive correlation, while the counts of white blood cells and platelets, as well as the levels of albumin and globulin, demonstrated a significant negative correlation.

### Diagnostic performance and model development

The parameters that exhibited significant differences between the two groups showed only intermediate discriminatory ability in the ROC analysis (Table 3). SUVmax-lymph nodes/Mediastinum had the highest AUC (0.794), with an optimal cutoff of > 4.9, and corresponding sensitivity and specificity values of only 66.7% and 77.4%, respectively. IFN-γ exhibited the highest sensitivity at 90.9%, while its specificity was 56.5%. EBV DNA copies in plasma > 35,000 demonstrated the highest specificity at 89.3%, while its sensitivity was 61.5%.

**Fig. 4:**
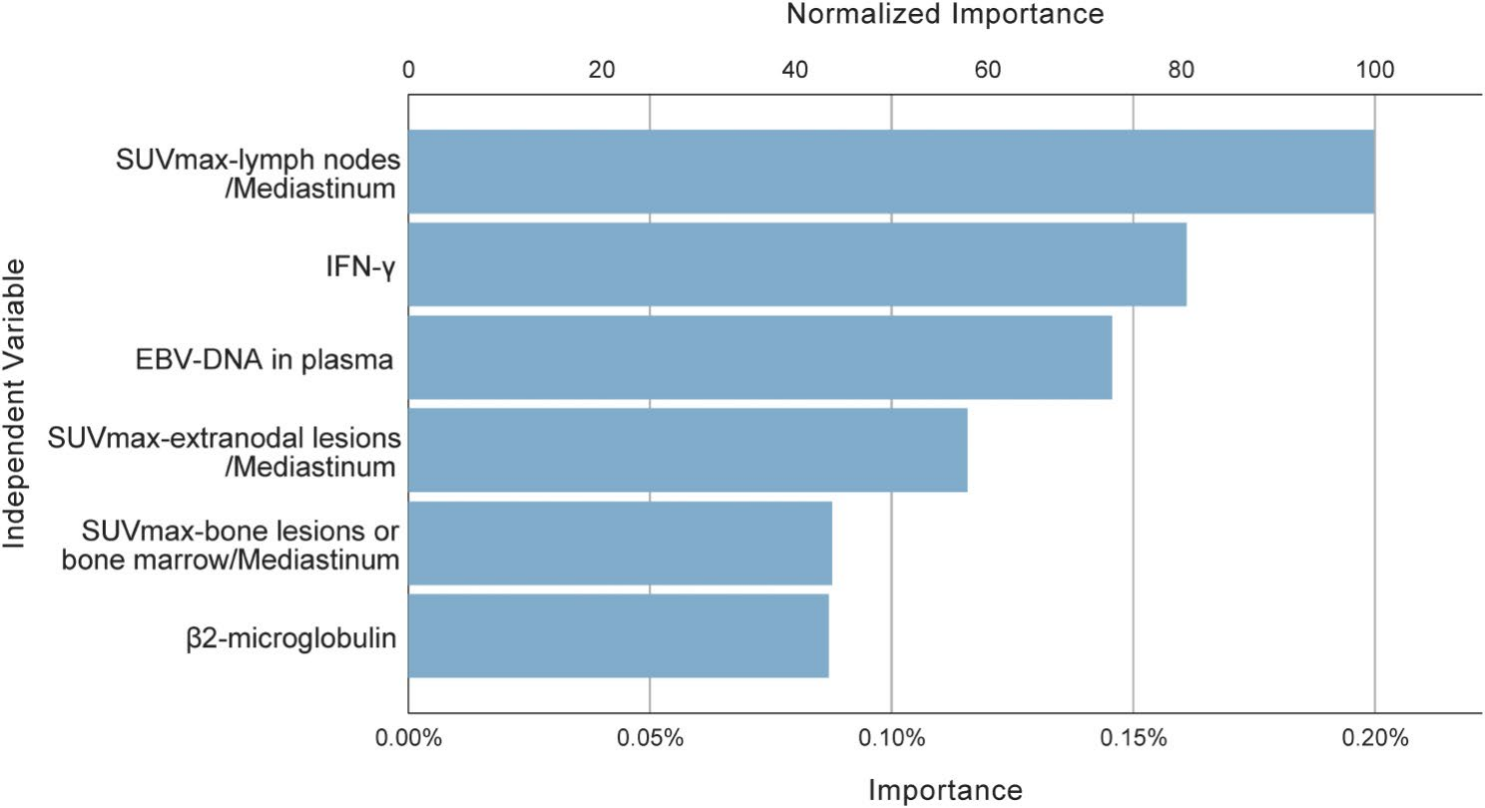
The important variables selected based on Gini index in CART analysis to differentiate EBV-positive LA-HLH patients with EBV-HLH patients. The importance is a measure of how much the variable contributes to the differential diagnosis. Normalized importance is calculated by dividing the importance values by the largest one and is expressed as percentages

To established an unbiased and practical diagnostic rule, we conducted CART analysis with cross-validation, considering all significant parameters from the univariate analysis. Ultimately, six variables were identified to have the greatest effect on distinguishing LA-HLH among HLH patients with EBV infection: SUVmax-lymph nodes/Mediastinum, IFN-γ, EBV-DNA in plasma, SUVmax-extranodal lesions/Mediastinum, SUVmax-bone lesions or bone marrow/Mediastinum, and β2-microglobulin. The importance of these variables is depicted in Fig. 4. The decision tree is shown in supplementary Fig. 1.

**Fig. 5:**
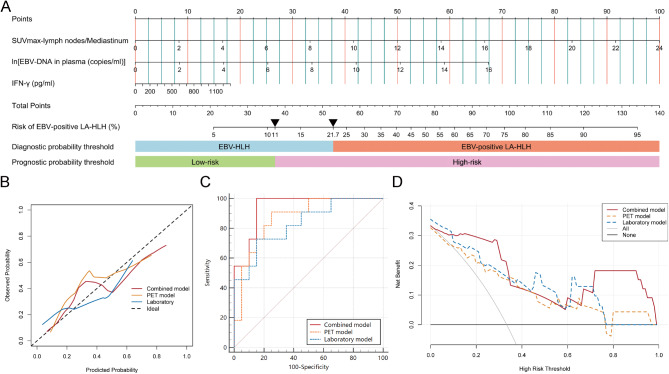
Nomogram of the combined model and the evaluation of the models. Two triangles are annotated on the nomogram to represent the thresholds for diagnosis and prognosis respectively (**A**). The calibration curves (**B**), receiver operating characteristic curves (**C**), and the decision curves analysis (**D**) of the models

Logistic regression analyses were performed on the important PET parameters and laboratory parameters to develop PET model (supplementary Fig. 2A), laboratory model (supplementary Fig. 2B), and combined model (Fig. 5A), respectively. The combined model incorporated SUVmax-lymph nodes/Mediastinum, EBV-DNA in plasma and IFN-γ, yielding an AUC of 0.926 (95%CI: 0.779–0.988). At an optimal threshold probability of 21.7%, the sensitivity achieved 100% and the specificity was 81.8%. The calibration curves and ROC curves were shown in Fig. 5B and C. The DCA curves indicated that the combined model provided the greatest net benefit in clinical usefulness within threshold probability ranges of 0.1 ~ 0.3 or 0.75 ~ 1.0 (Fig. 5D).

**Fig. 6:**
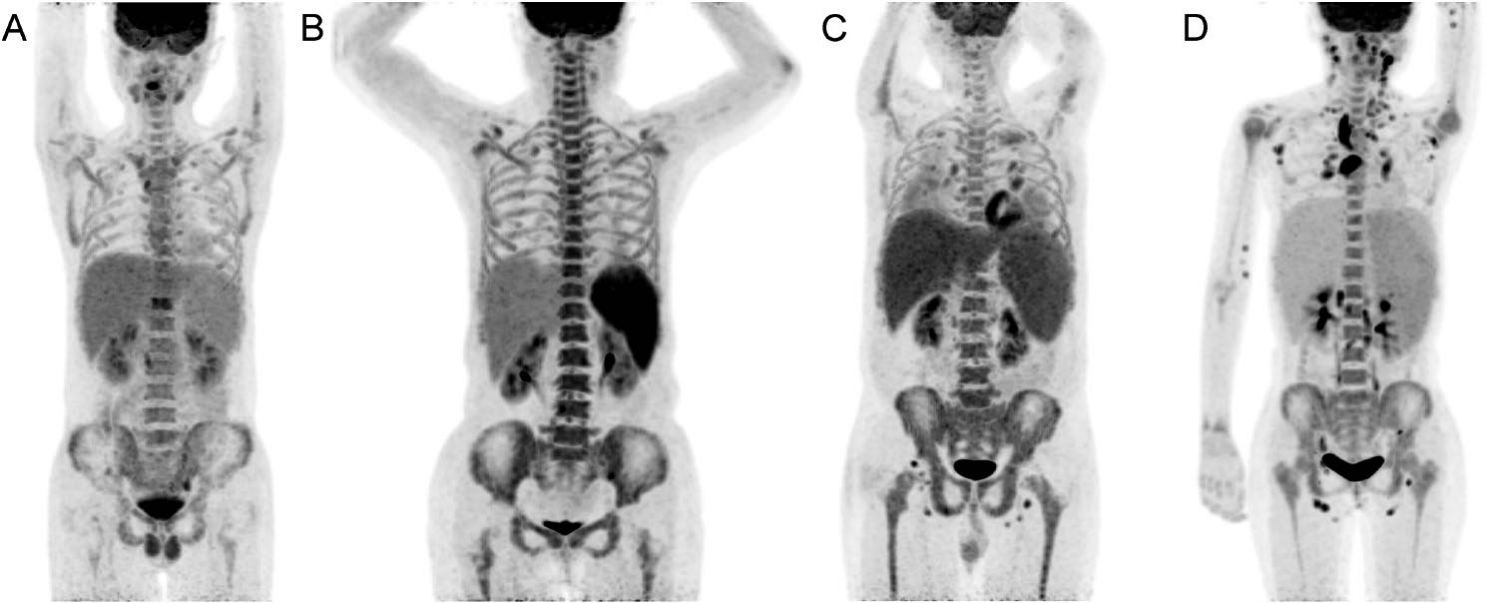
^18^F-FDG PET/CT maximum intensity projection images depicting four HLH patients with EBV infection, wherein the combined model effectively differentiated the cause. Focal hypermetabolic lesions of bone were observed in a 19-year-old male (**A**) and a 64-year-old female (**B**), but no lymphoma was found by bone puncture or lymph node biopsy. A 53-year-old male (**C**) and an 18-year-old female (**D**) showed only hypermetabolic lymph nodes with symmetrical distribution and were diagnosed with Hodgkin lymphoma and NK/T-cell lymphoma, respectively, based on lymph node biopsy

The Delong’s test for the AUC of the models indicated no significant difference between the combined model and the PET or laboratory model. Nevertheless, comparing the NRI and IDI between the models revealed a significant improvement in reclassification ability with the combined model (Table 3). Figure 6 illustrates four HLH patients with EBV infection in whom it was difficult to distinguish by visual assessment, yet the combined model enabled effective differentiation.

**Table 3:** Performance of each variable and multivariate models for the diagnosis of lymphoma in adult HLH patients with EBV infection

Variables	Optimized cutoffs	Sensitivity(%)	Specificity(%)	AUC	95%CI
^18^F-FDG PET/CT parameters					
SUVmax-lymph nodes/Mediastinum	> 4.9	66.7	77.4	0.794	0.649 ~ 0.899
SUVmax-bone lesions or bone marrow/Mediastinum	> 2.5	80.0	64.5	0.705	0.553 ~ 0.830
SUVmax-extranodal lesions/Mediastinum	> 4.7	66.7	83.9	0.733	0.582 ~ 0.853
SUVmax-Spleen/Mediastinum	> 1.9	71.4	67.7	0.707	0.553 ~ 0.833
PPD, cm^2^	> 1.4	73.3	71.0	0.743	0.593 ~ 0.860
Laboratory parameters					
β2-microglobulin, mg/L	> 4.1	76.9	68.0	0.702	0.532 ~ 0.839
EBV-DNA in plasma (copies/ml)	> 35,000	61.5	89.3	0.760	0.601 ~ 0.879
IFN-γ	> 60	90.9	56.5	0.711	0.531 ~ 0.853
Multivariate models					
PET model (*n* = 46)	> 17.7%	86.7	67.7	0.830	0.690 ~ 0.925
laboratory model (*n* = 31)	> 45.7%	72.7	85.0	0.832	0.655 ~ 0.941
Combined model (*n* = 41)	> 21.7%	100.0	81.8	0.926	0.779 ~ 0.988
Compare the models	NRI (95%CI)	*P* value	IDI (95%CI)	*P* value	Delong’s test (*Z*, *P* value)
Combined model vs. PET model	0.955 (0.306 ~ 1.604)	0.004**	0.158 (0.021 ~ 0.294)	0.024*	1.134, 0.257
Combined model vs. Laboratory model	0.773 (0.089 ~ 1.456)	0.027*	0.252 (0.038 ~ 0.465)	0.021*	1.411, 0.158

PPD = Products of the longest perpendicular diameters; EBV = Epstein-Barr Virus; IFN = interferon; NRI = Net Reclassification Index; IDI = Integrated Discrimination Improvement; AUC = Area Under the Curve

*Significance at *P* < 0.05. **Significance at *P* < 0.01

### Prognostic prediction by the combined model

**Fig. 7:**
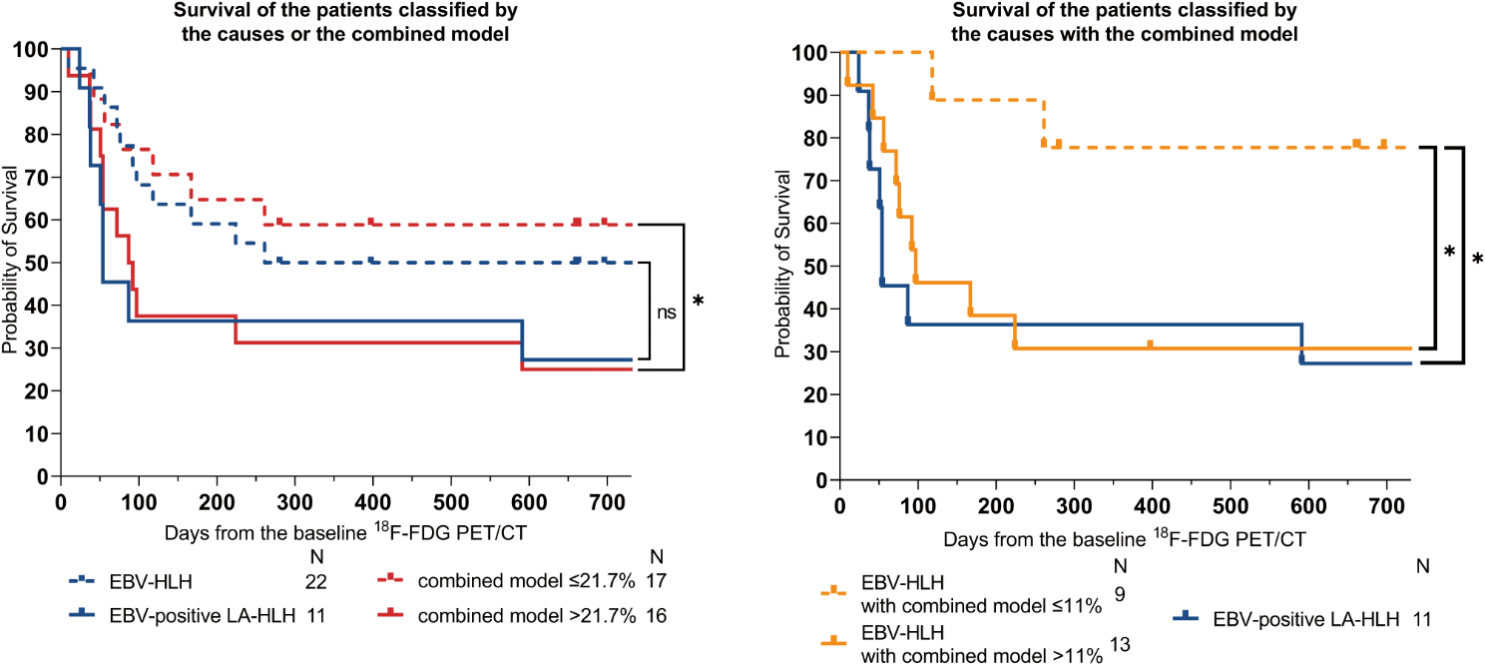
The combined model is highly predictive of mortality in HLH patients with EBV infections. The Kaplan-Meier curves of patients classified by lymphoma pathological diagnosis or the combined model (**A**). The Kaplan-Meier curves of EBV-HLH patients further classified by the combined model (**B**)

Patients were grouped according to the underlying causes and the diagnostic threshold of the combined model, respectively, and their respective Kaplan-Meier curves were illustrated in Fig. 7A. No statistically significant difference in prognosis was observed between EBV-positive LA-HLH and EBV-HLH, whereas patients with a positive combined model (probability >21.7%) had a significantly poorer prognosis compared to those with a negative combined model (probability ≤ 21.7%). We further stratified EBV-HLH patients into two groups using a prognostic cut-off probability of the combined model determined via running log-rank tests (Fig. 7B). For EBV-HLH patients without lymphoma, those with a combined model probability > 11% had a significantly worse prognosis compared to patients with a combined model probability ≤ 11% (22 patients; HR, 4.2; 95%CI, 1.3–13.8), and their prognosis resembled that of patients with LA-HLH. Among the 13 high-risk EBV-HLH patients with a combined model probability > 11%, 4 (30.8%) experienced rapid disease progression resulting in death, 7(53.8%) patients were subsequently diagnosed with chronic active EBV infection during follow-up (of whom 5 died), and 2 patients achieved stabilization following induction chemotherapy.

Notably, analysis of EBV-infected lymphocyte populations in 10 high-risk EBV-HLH patients revealed that 9 were T- or NK/NKT- cell EBV-positive (90%), compared to only 2 out of 8 low-risk EBV-HLH patients (25%). This difference was statistically significant (*P* = 0.013). In contrast, there was no difference in B-cell EBV positivity between the two groups.

## Discussion

In this retrospective study of 46 HLH patients with EBV infection, we evaluated the discriminative ability of ^18^F-FDG PET/CT for potential lymphoma detection. Among these patients, EBV-positive LA-HLH cases exhibited heightened metabolic activity in lymph nodes, bone lesions or bone marrow, spleen, and other extranodal lesions when compared to EBV-HLH patients without detectable lymphoma. Nevertheless, the similarity in imaging presentations and substantial overlap in metabolic activity limit effective differentiation. By integrating laboratory parameters, including plasma EBV-DNA and IFN-γ, we constructed a multivariate model that significantly improved differential accuracy and offered better predictive value for poor prognosis compared to the lymphoma diagnosis.

EBV-associated lymphoproliferative disorders (EBV-LPD) encompass a broad clinicopathological spectrum ranging from self-limiting polymorphic reactive proliferations to highly aggressive B-cell or T-cell lymphomas [18]. Moreover, all diseases within this spectrum could be accompanied by HLH [19]. Clonal gene rearrangement of IG and TCR serve as specific markers for monoclonal hyperplasia in B and T lymphocytes, and can be used to distinguish between benign or malignant lymphocytes [20, 21]. However, in EBV-LPD, the presence of monoclonal IG and TCR gene rearrangements doesn’t inherently indicate malignancy; these rearrangements are observed irrespective of the patient’s progression to lymphoma [18]. In this study, the clonal gene rearrangement was observed in both EBV-HLH and EBV-positive LA-HLH patients. EBV predominantly infects B-lymphocytes, leading to a higher prevalence of B-cell-derived EBV-LPDs compared to those originating from T/NK-cells. Nevertheless, EBV associated T/NK-cell LPDs are often accompanied by HLH, which may be due to an excessive pro-inflammatory response of the infected T/NK cells [22]. This explains the highest percentage of Peripheral T-cell lymphoma or NK/T-cell lymphoma in this study.

The prominent ^18^F-FDG PET/CT findings in HLH may be attributed to the hyperactivation of T cells and macrophages. These findings include hepatosplenomegaly with diffusely increased FDG uptake; diffusely increased FDG uptake in the axial and appendicular skeleton; hypermetabolic lymphadenopathy; and serous effusions [23, 24]. In adult sHLH, a small sample study was performed to evaluated the diagnostic performance of ^18^F-FDG PET/CT in distinguishing between non-malignancy and malignancy associated HLH. The sensitivity and specificity were 83.0% and 62.5% respectively [25]. Additionally, the SUVmax values of the spleen, bone marrow, and lymph nodes were higher in malignancy associated HLH compared to non-malignancy associated HLH [11, 26]. Moreover, a multivariate diagnostic model incorporating metabolic parameters and age was developed and internally validated. The model achieved a sensitivity of 90.0, specificity of 68.8, and an AUC of 0.875 in the validation set [11]. However, the lymphoid hyperplasia due to EBV infection makes the differential diagnosis more difficult, and the diagnostic efficacy of ^18^F-FDG PET/CT in distinguishing between EBV-HLH, systemic chronic active EBV infection (CAEBV), and EBV-positive T/NK-cell lymphomas remains undefined [22]. Lu et al. analysed ^18^F-FDG PET/CT images in 29 pediatric HLH patients with EBV infection and found that multi-organ extranodal lesions were more frequently observed in malignancy-associated HLH, whereas the extranodal lesions in non-malignancy-associated HLH generally involved a single organ, even though the involved organs were diverse, including bone marrow, spleen, adrenal gland, or muscles [27]. But in this study, there was no statistically significant difference in the frequency of either single-organ or multi-organ extranodal lesions between the two groups, and the difference would be further reduced if the bone lesions were excluded. The study mentioned above observed significantly higher SUVmax-lymph nodes/mediastinum in patients with malignancy-associated HLH, which is consistent with our present findings. Additionally, in our study, differences in SUVmax ratios of spleen, bone lesions or bone marrow were also noted between the two groups. It has been reported that malignancy-associated HLH tends to occur in relatively older individuals, whether in children or adults with sHLH [11, 27]. However, we found no significant age difference between the two groups among adult HLH patients with EBV infection.

In terms of laboratory examinations, higher levels of temperature, serum β2-microglobulin levels, serum IFN-γ levels, and EBV DNA copies in plasma were observed in EBV-positive LA-HLH patients. Both higher body temperature and IFN-γ indicated a higher inflammatory response. IFN-γ is the one of the most critical cytokines in the cytokine storm associated HLH. It is released by activated CD4 + T cells, CD8 + T cells, NK cells, and dendritic cells, further stimulating inflammatory cells like macrophages, monocytes, and neutrophils. Additionally, IFN-γ can reciprocally activate CD4 + T cells and dendritic cells [28]. The β2- microglobulin is a single polypeptide chain found on all cell membranes linked to MHC class I cell surface antigens. It is prominently released into the bloodstream during systemic inflammation and hematologic malignancies, correlating with increased tumor burden and unfavorable prognosis [29]. Jiang et al. reported higher levels of β2- microglobulin in lymphoma-associated HLH compared to benign disease-associated HLH, consistent with our study [30]. EBER positivity has been reported to correlate with a higher plasma EBV-DNA load, both indicators of a more severe condition and a poorer prognosis [31, 32]. In this study, EBV-positive LA-HLH patients showed higher plasma EBV-DNA levels and a greater proportion of EBER positivity, which may suggest a prolonged and more severe EBV infection in these individuals.

Compared to the SUVmax of lymph nodes, the SUVmax of bone lesions or bone marrow and the SUVmax of the spleen exhibited stronger correlations with more laboratory examinations including body temperature, C-reactive protein, serum ferritin, β2-microglobulin, sCD25, IL-1Ra, IL-8, IL-18, GM-CSF, and TNF-α, reflecting a higher intensity of inflammatory activity. The positive correlation with EBV-DNA in plasma can be attributed to the heightened inflammatory response induced by elevated EBV loads. The negative correlation with blood routine examinations (white blood cells, hemoglobin, and platelets), albumin, and globulin indicates that the metabolism of bone marrow and spleen may signify hyperactive hematopoietic activity due to hemocytopenia, alongside hepatic injuries, and immune dysregulation resulting from an intense inflammatory response [24]. Therefore, the metabolism activity observed in the bone and spleen is influenced by multiple factors and might demonstrated lower specificity compared to that observed in lymph nodes.

After conducting decision tree analysis and multivariate regression, the SUVmax-lymph nodes/mediastinum, EBV-DNA in plasma, and IFN-γ were selected to construct the combined model. The NRI and IDI quantify the model’s enhanced differential diagnostic capability, complementing the limitations of relying solely on AUC for assessing model performance [33]. Based on these metrics, the combined model significantly improved discriminatory ability compared to models using only PET or laboratory parameters. It effectively distinguishes EBV-HLH patients who have focal lesions with moderately increased FDG uptake. However, limited by the non-specific property of the lymph nodes metabolism, the combined model still has a number of “false-positive” cases. Our findings indicated that despite the absence of lymphoma in these cases classified as “false-positive”, the prognosis of these patients is equally unfavorable compared to those with LA-HLH. Therefore, it is imperative to consider the likelihood of an occult malignancy in these patients and administer aggressive treatment. The large number of hypermetabolism foci on ^18^F-FDG PET/CT in HLH patients with EBV infection may portend CAEBV with refractory HLH. We found that nomogram defined high-risk EBV-HLH was associated with EBV + T or NK/NKT- cell. It has been reported that EBV + T cells may result from EBV infection of haematopoietic stem cells, which ultimately requires allogeneic hematopoietic stem cell transplantation for cure [34].

Our study has several limitations. Firstly, it is a retrospective study, which may lead to possible selection bias, and the uneven distribution of patients between the two groups is a concern. The diversity of pathological subtypes within the EBV + LA-HLH group may have influenced the results. Secondly, the rarity of the disease and the requirement for a pre-treatment ^18^F-FDG PET/CT examination in this study resulted in a small sample size, and a separate validation cohort is needed. Thirdly, our results did not reveal a statistically significant difference in prognosis between LA-HLH patients and those with non-malignancy associated HLH, which is inconsistent with the findings of previous studies [4, 35, 36]. This discrepancy might be attributed to the poor prognosis among some EBV-HLH patients and the small sample size in this study. Fourthly, the line between neoplastic and non-neoplastic EBV-HLH is blurred based on clinical and pathologic criteria, the differentiation is challenging [22]. The grouping of patients in this study relied to some extent on the empirical assessment of pathologists and clinicians, potentially affecting the accuracy of the results.

## Conclusions

In this study, the integration of ^18^F-FDG PET/CT parameters with laboratory examinations enhances the ability to differentiate between EBV-HLH and EBV-positive LA-HLH, despite the inevitability of false positives, this combined diagnostic approach provides valuable insights into patient prognosis and can guide more targeted treatment strategies. Future studies with larger cohorts and prospective designs are necessary to validate these findings and refine diagnostic models.

### Electronic supplementary material

Below is the link to the electronic supplementary material.

Supplementary Material 1

## Data Availability

The datasets generated during and/or analysed during the current study are available from the corresponding author on reasonable request.

## Abbreviations

HLH: Hemophagocytic lymphohistiocytosis
EBV: Epstein-Barr virus
sHLH: Secondary HLH
LA-HLH: Lymphoma-associated HLH
LPD: Lymphoproliferative disorders
EBER: EBV-encoded small RNA
OS: Overall survival
SUV: Standardized uptake value
VOI: Volume of interest
DCA: Decision curve analysis
ROC: Receiver-Operating-Characteristic
AUC: Area under the curve
NRI: Net Reclassification Index
IDI: Integrated Discrimination Improvement
HR: Hazard ratio
CI: Confidence interval
IGH: Immunoglobulin H
IGK: Immunoglobulin kappa
IGL: Immunoglobulin lambda
TCRB: T cell receptor beta
TCRD: T cell receptor delta
TCRG: T cell receptor gamma
CAEBV: Chronic active EBV infection
FDG: Fluorodeoxyglucose
PET: Positron emission tomography
CT: Computed tomography
