# Supplementary material for: Enhancing diagnostic precision in EBV-related HLH: a multifaceted approach using 18F-FDG PET/CT and nomogram integration
Source: Cancer Imaging. 2024 Aug 18;24:108. doi: 10.1186/s40644-024-00757-w (PMC11330599; doi:10.1186/s40644-024-00757-w)
Supplement: Supplementary file 1 — Supplementary Material 1 [file 40644_2024_757_MOESM1_ESM.docx]

Supplemental Figure. 1


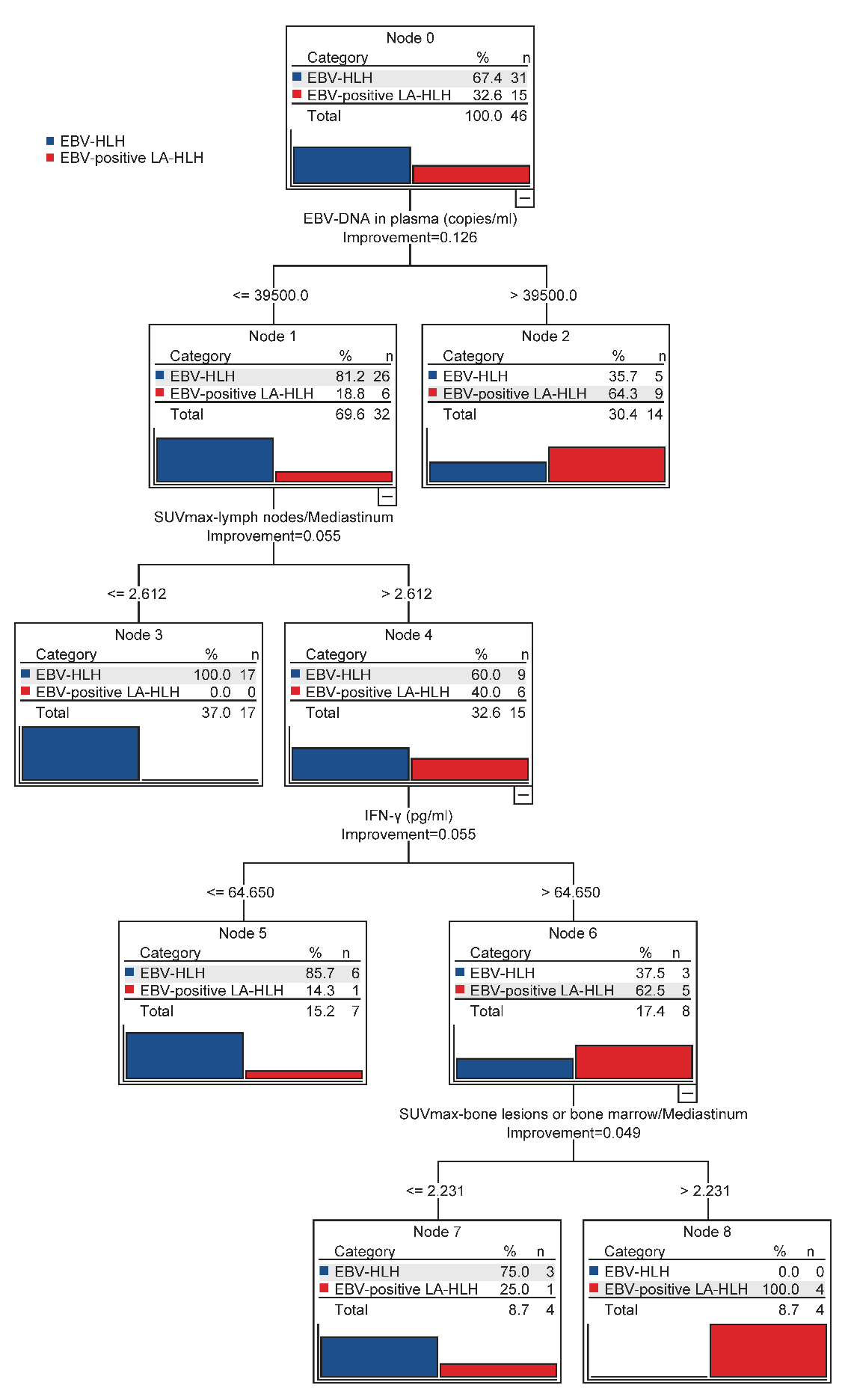


Supplemental Fig. 1 A decision tree employing the CART growing method

Important variables were selected based on the Gini index, with a minimum of 4 cases for parent nodes and a minimum of 3 cases for child nodes. The minimum change in improvement was defined as 0.0001, and pruning tree to avoid overfitting with the maximum difference in risk of 1 standard error. Due to the small sample size and pruning, β2-microglobulin and SUVmax-extranodal lesions/Mediastinum were not included in the decision tree.

Supplemental Figure. 2


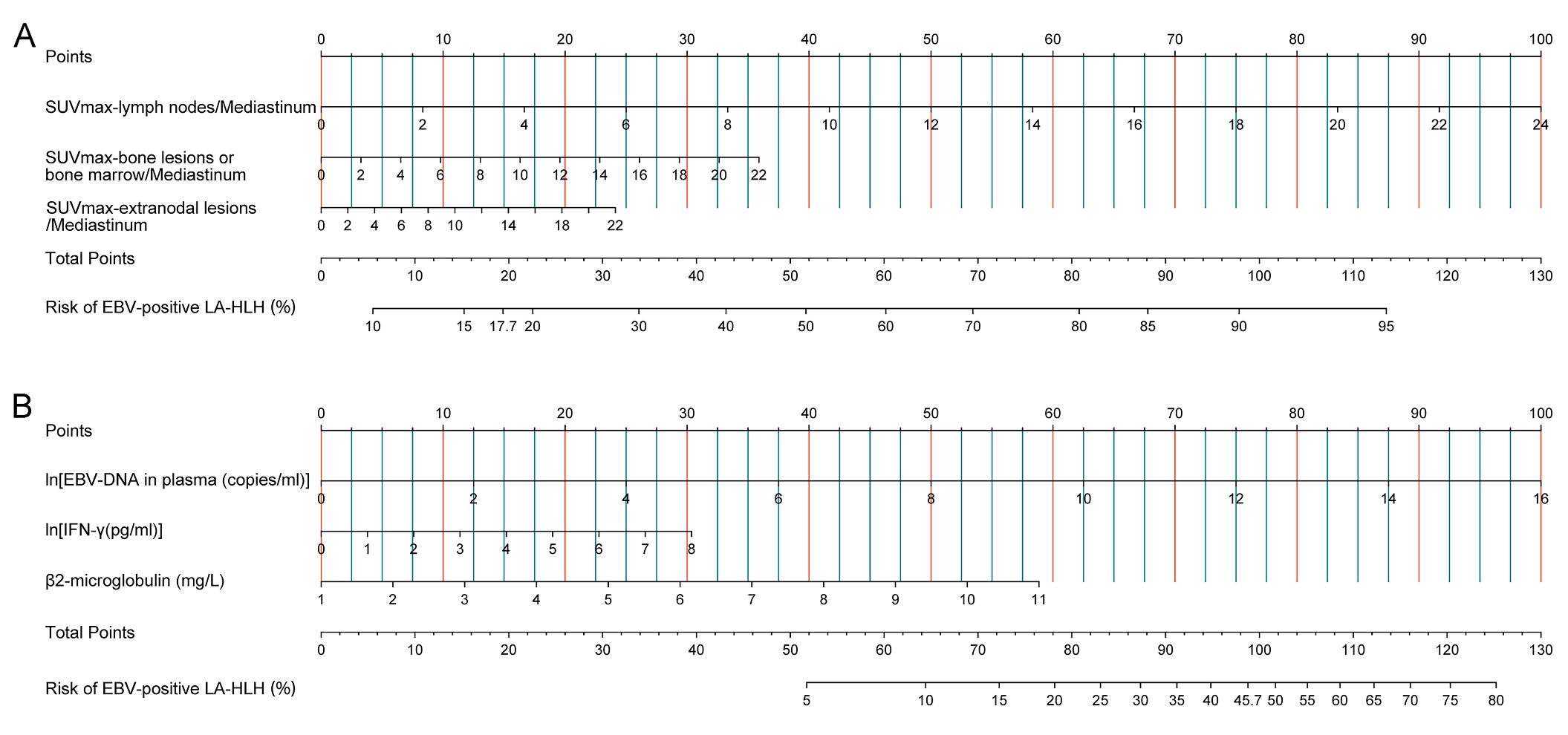


Supplemental Fig. 2 The nomograms of the PET model (A) and laboratory model (B) constructed by logistic regression analyses

Supplemental Table 1. Baseline laboratory examinations in adult HLH patients with EBV infection

|  | EBV-HLH (n=31) | |  | EBV-positive LA-HLH (n=15) | |  |
| --- | --- | --- | --- | --- | --- | --- |
| Variables | N | Summary measure |  | N | Summary measure | *P* value |
| Physical sign |  |  |  |  |  |  |
| Temperature, ℃ | 27 | 37 (36.7, 37.9) |  | 12 | 38.4 (37.3, 38.9) | 0.036^*^ |
| Laboratory parameters |  |  |  |  |  |  |
| WBC, ×10^9^/L | 30 | 3.85 (1.37, 5.55) |  | 14 | 2.71 (1.25, 5.16) | 0.450 |
| ANC, ×109/L | 30 | 1.87 (0.73, 3.79) |  | 14 | 1.43 (0.66, 2.72) | 0.623 |
| HGB, g/L | 30 | 97 (86, 119) |  | 14 | 72 (65, 109) | 0.091 |
| PLT, ×10^9^/L | 30 | 93 (50, 196) |  | 14 | 83 (36, 187) | 0.801 |
| CRP, mg/L | 30 | 14.0 (2.0, 41.8) |  | 14 | 17.6 (10.5, 54.9) | 0.140 |
| ALT, U/L | 29 | 56.0 (30.5, 78.0) |  | 13 | 49.0 (21.5, 109.0) | 0.979 |
| AST, U/L | 29 | 45.7 (25.0, 98.7) |  | 13 | 94.2 (27.0, 139.6) | 0.214 |
| Albumin, g/L | 29 | 31.9±5.4 |  | 13 | 30.2±5.0 | 0.322 |
| Globulin, g/L | 29 | 29.3±7.5 |  | 13 | 28.0±11.9 | 0.722 |
| TG, mmol/L | 29 | 1.89 (1.54, 2.52) |  | 13 | 2.00 (1.36, 3.78) | 0.707 |
| BUN, mmol/L | 29 | 4.12 (3.38, 5.16) |  | 13 | 4.13 (3.02, 7.74) | 0.768 |
| Creatinine, μmol/L | 29 | 57.1 (47.2, 66.6) |  | 13 | 50.6 (38.9, 57.4) | 0.143 |
| SF, ng/ml | 29 | 1095.7 (404.0, 1844.4) |  | 13 | 1974.3 (957.4, 5475.5) | 0.159 |
| FBG, g/L | 29 | 2.01 (1.41, 3.15) |  | 13 | 2.74 (0.99, 3.77) | 0.957 |
| Procalcitonin, ng/ml | 28 | 0.3 (0.18, 0.53) |  | 13 | 0.45 (0.23, 1.01) | 0.353 |
| ESR, mm/h | 29 | 18.0 (9.0, 35.0) |  | 13 | 36.0 (9.5, 54.5) | 0.318 |
| LDH, U/L | 29 | 346.0 (207.5, 667.5) |  | 13 | 516.0 (414.5, 719.0) | 0.077 |
| β2-microglobulin, mg/L | 25 | 3.61 (2.63, 4.58) |  | 13 | 4.30 (4.05, 5.48) | 0.044^*^ |
| sCD25 | 28 | 12620 (4286, 32086) |  | 13 | 31303 (5277, 44000) | 0.135 |
| sCD25/SF | 28 | 9.87 (3.18, 34.43) |  | 13 | 13.06 (1.65, 30.62) | 1.000 |
| NK cell activity (%) | 24 | 16.1 (15.2, 18.5) |  | 13 | 15.3 (13.1, 17.1) | 0.179 |
| Inflammatory cytokines (pg/ml) |  |  |  |  |  |  |
| IL-1α | 22 | 0.4 (0.3, 0.8) |  | 11 | 0.4 (0.3, 3.3) | 0.248 |
| IL-1β | 22 | 0.80 (0.7, 1.6) |  | 11 | 1.2 (0.9, 4.8) | 0.233 |
| IL-1RA | 22 | 124.4 (28.0, 703.7) |  | 11 | 849.2 (121.3, 1995.0) | 0.166 |
| IL-2 | 22 | 3.5 (2.8, 6.5) |  | 11 | 3.5 (2.4, 4.2) | 0.560 |
| IL-4 | 22 | 5.6 (4.7, 7.7) |  | 11 | 5.8 (2.2, 24.9) | 0.985 |
| IL-6 | 23 | 5.2 (4.2, 8.1) |  | 11 | 7.2 (4.9, 40.3) | 0.513 |
| IL-8 | 23 | 1.4 (1.1, 36.0) |  | 11 | 5.8 (2.8, 15.1) | 0.258 |
| IL-10 | 23 | 7.6 (1.3, 43.5) |  | 11 | 27.1 (6.8, 67.1) | 0.274 |
| IL-12 | 22 | 3.4 (2.5, 6.1) |  | 11 | 3.4 (2.9, 5.1) | 0.895 |
| IL-17 | 23 | 0.9 (0.5, 1.3) |  | 11 | 1.3 (1.0, 4.3) | 0.114 |
| IL-18 | 23 | 163.8 (53.9, 483.6) |  | 11 | 168.1 (60.7, 359.1) | 0.800 |
| IL-23 | 22 | 6.6 (4.5, 8.6) |  | 11 | 8.6 (6.5, 14.0) | 0.089 |
| IFN-α | 22 | 0.2 (0.2, 0.3) |  | 11 | 0.2 (0.1, 0.3) | 0.178 |
| IFN-γ | 23 | 54.5 (21.8, 125.7) |  | 11 | 190.4 (69.3, 262.4) | 0.050 |
| GM-CSF | 22 | 6.6 (4.8, 8.1) |  | 11 | 6.6 (5.2, 7.6) | 0.925 |
| TNF-α | 23 | 5.8 (4.9, 18.1) |  | 11 | 7.5 (4.5, 22.7) | 0.663 |
| EBV-DNA |  |  |  |  |  |  |
| EBV-DNA in plasma (copies/ml) | 28 | 500 (500, 21500) |  | 13 | 59000 (1504, 315000) | 0.007^**^ |
| EBV-DNA in PBMC (copies/ml) | 27 | 5500 (500, 43000) |  | 14 | 9100 (500, 31790) | 0.674 |

Some number is confined to patients who underwent each test.

WBC=White blood cell; ANC=Absolute neutrophil count; HGB=Hemoglobin; PLT=Platelet count; CRP=C-reactive protein; ALT=Alanine aminotransferase; AST=Aspartate aminotransferase; TG=Triglycerides; BUN=Blood urea nitrogen; SF=serum ferritin; FBG=fibrinogen; ESR=erythrocyte sedimentation rate; LDH=lactate dehydrogenase; sCD25=soluble interleukin-2 receptor (sIL-2R); NK=natural killer; EBV=Epstein-Barr Virus; PBMC=peripheral blood mononuclear cell; IL=interleukin; IFN=interferon; GM-CSF=granulocyte-macrophage colony stimulating factor; TNF=tumor necrosis factor;

Data are mean ± SD, median (25%, 75%), or number (%).

*Significance at P<0.05. **Significance at P<0.01.
